# Spontaneous Intestinal Perforation: An Atypical Presentation of Neutropenic Enterocolitis—A Case Report

**DOI:** 10.1155/2014/925078

**Published:** 2014-03-06

**Authors:** Aylin Canbolat Ayhan, Cetin Timur, Ersin Bocu, Neslihan Gulcin

**Affiliations:** ^1^Pediatric Hematology-Oncology Department, Goztepe Education and Research Hospital, Istanbul Medeniyet University, 34722 Istanbul, Turkey; ^2^Pediatric Surgery Department, Goztepe Education and Research Hospital, Istanbul Medeniyet University, 34722 Istanbul, Turkey

## Abstract

*Background*. Neutropenic enterocolitis is one of the most common gastrointestinal complications seen in patients who are receiving chemotherapy for leukemia. Severe neutropenia is the main underlying factor of this pathology. It is characterized by fever and abdominal pain. *Case Presentation*. Herein, we report a case of neutropenic enterocolitis which presented with intestinal perforation in an afebrile patient who was diagnosed with acute lymphoblastic leukemia and was receiving induction chemotherapy. *Conclusion*. We aimed to emphasize the importance of enterocolitis and increase awareness against such severe complications which could have unexpected presentations.

## 1. Introduction

Neutropenic enterocolitis or typhlitis is characterized by fever and abdominal pain [1]. It is seen primarily in severely immunosuppressed and neutropenic patients with leukemia [1, 2]. Herein, we report a case of neutropenic enterocolitis which presented with spontaneous intestinal perforation in an afebrile patient who was receiving induction chemotherapy for acute lymphoblastic leukemia.

## 2. Case

**Figure 1:**
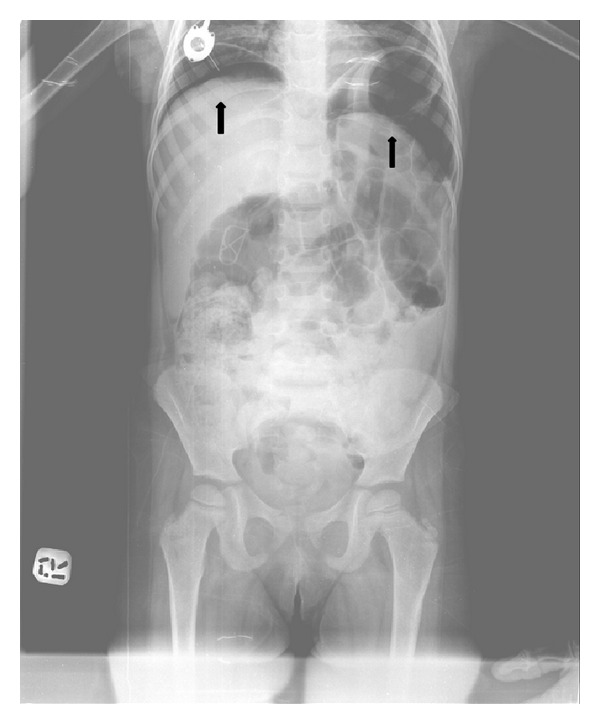
Plain abdominal radiograph shows subdiaphragmatic free air.

**Figure 2:**
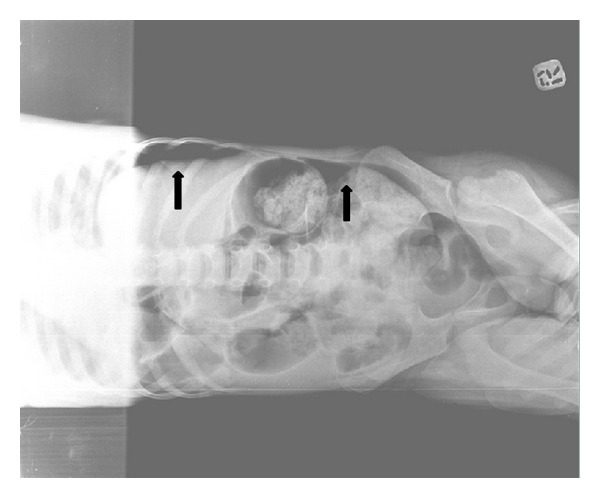
Lateral decubitus radiograph shows air movement within abdominal cavity.

Our patient was a 4-year-old girl diagnosed with common acute lymphoblastic leukemia. She was on remission induction phase of chemotherapy consisting of prednisolone (60 mg/m^2^/day; 1–28 days; from day 29 tapered to withdrawal over 9 days), vincristine (1.5 mg/m^2^/day; days 8, 15, 22, and 29), L-asparaginase (5,000 U/m^2^/day; days 12, 15, 18, 21, 24, 27, 30, and 33), daunorubicin (30 mg/m^2^/day; days 8 and 15), and intrathecal methotrexate (dosage adjusted to age). She was in deep neutropenia since she was admitted to hospital. On the day 27 of chemotherapy, she developed fever. Her leukocyte count was 0.5  × 10^9^/L and absolute neutrophil count was 0.0  × 10^9^/L. C-reactive protein (CRP) was 2.6 mg/dL. Her renal, liver function panel, and urinalysis were normal. Blood from the port catheter and pheripheral vein was sampled for cultures and then broad-spectrum antibiotics were initiated empirically and chemotherapy was postponed. On her physical examination there was not any focus on fever. Her oral mucous membrane was normal. She did not have oral mucositis. Her liver and spleen were not palpable. She did not have abdominal tenderness, distension, or any other clinical findings related to abdominal distress. Her oral intake was good. She did not have vomiting and her stool was normal. Thorax radiograph was normal. Her fever lasted for two days. On the 3rd day of antibiotics her fever resolved; on the 4th day she was still afebrile but she developed abdominal pain. Physical examination revealed abdominal distension and tenderness. Abdominal ultrasonography was performed but, because of the presence of free intense air superposition, the bowels were not evaluated optimally. Plain abdominal radiograph demonstrated subdiaphragmatic free intra-abdominal air (Figure 1) and the one obtained in lateral decubitus revealed free air movement within the abdominal cavity (Figure 2). She was operated immediately. During operation it was seen that the small intestine was perforated from approximately 2.5 cm apart from cecum. Small intestine and colon were very fragile, inflammatory, and bloody. Necrotic tissue was removed completely. Pathological analysis of the specimen revealed findings related to chronic inflammatory degenerative changes in intestine. It excluded malignancy. After surgery we treated her with bowel rest and decompression using nasogastric tube, total parenteral nutrition, and broad-spectrum antibiotics. Chemotherapy was postponed until complete recovery. On the day 15 of surgery, she was in good condition; her physical examination and laboratory findings were normal and we decided to continue chemotherapy.

## 3. Discussion

Enterocolitis of the ileocecal region in neutropenic patients is called typhlitis [1, 3]. It is associated with neutropenia, impaired host defense to invasion by microorganisms, and mucosal injury by chemotherapy agents [1–3]. Cytotoxic drugs may also cause ischemic cecal mucosal injury [4]. Corticosteroids, cytosine arabinoside, vincristine, cylophosphamide, irinotecan, cisplatin, and daunomycin are some of the chemotherapy agents which are found to be related to typhlitis [1, 4–6]. Our patient was neutropenic since she was admitted to hostipal and she was receiving prednisolone for 27 days. Also she received vincristine, daunorubicin, and L-asparaginase according to the chemotherapy regimen. In patients with leukemia, as a result of neutropenia or corticosteroids, proliferation of bacteria occurs secondary to decreased host defenses [1]. Marie et al. have reported a case of typhlitis after alemtuzumab which is very rare [7]. The pooled incidence rate of neutropenic enterocolitis in patients with acute leukemia treated with chemotherapy is reported at 5.6% [1]. The mortality rate is very high ranging between 50% and 100% [8, 9]. Fever, abdominal pain especially in the right lower quadrant with or without rebound tenderness, abdominal distension, diarrhea, nausea, and vomiting in the patients with neutropenia are the main clinical findings of neutropenic enterocolitis [1]. Plain abdominal radiograph and ultrasound and computed tomography are the helpful imaging studies in diagnosis. In our case because of the emergency of the patient we could not screen the abdomen with computed tomography. Bowel wall thickening, dilated cecum, pericecal fluid, inflammatory changes in the soft tissue, presence of intramural edema and right lower-quadrant inflammatory mass, and inflammatory stranding in the mesenteric fat surrounding the bowel are the main abnormal findings of imaging studies [1, 3, 10]. Bowel wall thickening is a valuable prognostic factor. In Cartoni et al.'s study it is reported that a thickness of >5 mm in the bowel wall is abnormal and diagnostic of neutropenic enterocolitis and the degree of bowel wall thickening significantly correlated with the outcome of the patients [11]. For patients with significant complications such as clinical deterioration during medical therapy, gastrointestinal bleeding, perforation, and peritonitis, surgery is recommended [1, 3, 9].

Neutropenic enterocolitis or typhlitis is characterized by fever and abdominal pain. Interestingly our patient was afebrile on the day of the diagnosis of perforation. She was receiving antibiotics for the last 3 days and fever was noted only on the day when the antibiotics were initiated. On the day when she was febrile, her clinical findings were normal. She did not have abdominal pain, tenderness, or vomiting. She was able to eat and her stool was normal. Because of this, on the first day of fever, we did not consider enterocolitis as the origin of the fever and we did not perform abdominal ultrasonography. We performed abdominal ultrasonograpy on the day when perforation occurred but because of the presence of free intense air superposition the bowels were not evaluated optimally. On the other hand it is well known that ultrasonography is very helpful in diagnosis of thyphilitis. It reveals a target sign encircling mural thickening as a result of mucosal edema in ileum and cecum [11].

## 4. Conclusion

In conclusion, our findings underline that a diagnosis of thyplitis and perforation should be considered in severe neutropenic patients who are receiving intense chemotherapy for leukemia even if they are afebrile at that time. Absence of fever may not exclude diagnosis of neutropenic enterocolitis and perforation.

## References

[1] Davila ML (2006). Neutropenic enterocolitis. *Current Opinion in Gastroenterology*.

[2] Urbach DR, Rotstein OD (1999). Typhlitis. *Canadian Journal of Surgery*.

[3] Shahani L (2012). Typhlitis: a neutropenic complication. *BMJ Case Reports*.

[4] Schlatter M, Snyder K, Freyer D (2002). Successful nonoperative management of typhlitis in pediatric oncology patients. *Journal of Pediatric Surgery*.

[5] Gray TLV, Ooi CY, Tran D, Traubici J, Gerstle JT, Sung L (2010). Gastrointestinal complications in children with acute myeloid leukemia. *Leukemia and Lymphoma*.

[6] Ji EH, Kim YM, Kim SJ (2012). A case of typhlitis developed after chemotherapy with irinotecan and cisplatin in a patient with small cell lung carcinoma. *Tuberculosis and Respiratory Diseases*.

[7] Marie I, Robaday S, Kerleau JM, Jardin F, Levesque H (2007). Typhlitis as a complication of alemtuzumab therapy. *Haematologica*.

[8] Alt B, Glass NR, Sollinger H (1985). Neutropenic enterocolitis in adults: review of the literature and assessment of surgical intervention. *The American Journal of Surgery*.

[9] Wade DS, Nava HR, Douglass HO (1992). Neutropenic enterocolitis: clinical diagnosis and treatment. *Cancer*.

[10] Sloas MM, Flynn PM, Kaste SC, Patrick CC (1993). Typhlitis in children with cancer: a 30-year experience. *Clinical Infectious Diseases*.

[11] Cartoni C, Dragoni F, Micozzi A (2001). Neutropenic enterocolitis in patients with acute leukemia: prognostic significance of bowel wall thickening detected by ultrasonography. *Journal of Clinical Oncology*.

